# CsgI (YccT) Is a Novel Inhibitor of Curli Fimbriae Formation in *Escherichia coli* Preventing CsgA Polymerization and Curli Gene Expression

**DOI:** 10.3390/ijms24054357

**Published:** 2023-02-22

**Authors:** Kotaro Sano, Hiroaki Kobayashi, Hirotaka Chuta, Nozomi Matsuyoshi, Yuki Kato, Hiroshi Ogasawara

**Affiliations:** 1 Research Center for Advanced Science and Technology, Division of Gene Research, Shinshu University, 3-15-1 Ueda, Nagano 386-8567, Japan; 2 Department of Applied Biology, Graduated School of Science and Technology, Shinshu University, 3-15-1 Ueda, Nagano 386-8567, Japan; 3 Academic Assembly School of Humanities and Social Sciences Institute of Humanities, Shinshu University, Matsumoto 390-8621, Japan; 4 Institute for Fiber Engineering (IFES), Interdisciplinary Cluster for Cutting Edge Research (ICCER), Shinshu University, Tokida 3-15-1, Ueda, Nagano 386-8567, Japan; 5 Renaissance Center for Applied Microbiology, Shinshu University, Nagano-shi, Nagano 380-8553, Japan

**Keywords:** CsgA, curli, *Escherichia coli*, OmpR, biofilm, periplasmic protein

## Abstract

Curli fimbriae are amyloids—found in bacteria (*Escherichia coli*)—that are involved in solid-surface adhesion and bacterial aggregation during biofilm formation. The curli protein CsgA is coded by a *csgBAC* operon gene, and the transcription factor CsgD is essential to induce its curli protein expression. However, the complete mechanism underlying curli fimbriae formation requires elucidation. Herein, we noted that curli fimbriae formation was inhibited by *yccT*—i.e., a gene that encodes a periplasmic protein of unknown function regulated by CsgD. Furthermore, curli fimbriae formation was strongly repressed by CsgD overexpression caused by a multicopy plasmid in BW25113—the non-cellulose-producing strain. YccT deficiency prevented these CsgD effects. YccT overexpression led to intracellular YccT accumulation and reduced CsgA expression. These effects were addressed by deleting the N-terminal signal peptide of YccT. Localization, gene expression, and phenotypic analyses revealed that YccT-dependent inhibition of curli fimbriae formation and curli protein expression was mediated by the two-component regulatory system EnvZ/OmpR. Purified YccT inhibited CsgA polymerization; however, no intracytoplasmic interaction between YccT and CsgA was detected. Thus, YccT—renamed CsgI (curli synthesis inhibitor)—is a novel inhibitor of curli fimbriae formation and has a dual role as an OmpR phosphorylation modulator and CsgA polymerization inhibitor.

## 1. Introduction

In their natural environment, many bacterial species form a biofilm after attaching to a solid surface [1]. These biofilms are predominantly composed of major components of the bacteria extracellular matrix, including extracellular DNA, lipids, polysaccharides, and proteins [2,3]. Biofilms allow bacteria to grow and survive in conditions of stress [4].

The Gram-negative bacterium *Escherichia coli* and the related species *Salmonella* spp. produce amyloid fibers, known as “curli fimbriae,” which are the major protein component of the extracellular matrix [5,6]. Curli fimbriae promote bacterial adhesion to solid surfaces and aggregation at the initial stage of biofilm formation. They facilitate the formation of floating biofilms called “pellicles” that are produced at the air–liquid interface and biofilms on solid surfaces [7,8,9,10]. Therefore, curli fimbriae are considered functional amyloids that facilitate bacterial survival [11].

In *E. coli* and *Salmonella*, factors required for curli fimbriae formation are encoded by the following two adjacent, divergently oriented operons: *csgBAC* and *csgDEFG* [5,12]. The expression of the curli fimbriae constituents CsgA and CsgB is encoded by the *csgBAC* operon and induced by CsgD—a biofilm master regulator. Both CsgE and CsgF are chaperone-like proteins involved in the extracellular transport of CsgA and CsgB from the periplasmic space [13,14]. Specifically, after crossing the inner membrane through the Sec transport system, CsgA and CsgB are transported via CsgE and CsgF to the outer membrane transporter CsgG; through this transporter, CsgA and CsgB are exported to the extracellular space [15]. On the cell surface, CsgB serves as an aggregation nucleus for CsgA and is important to anchor CsgA fibrils [12,15]. Accumulation of amyloid fibrils such as curli fimbriae in cells is cytotoxic and even lethal [16,17,18]. Factors that inhibit CsgA aggregation in *E. coli* have been identified, including chaperone proteins such as DnaK, Hsp33, and Spy [19]. CsgD induces the expression of CsgC; additionally, CsgC has been reported to efficiently transport CsgA to the extracellular space as a monomeric precursor protein of curli fimbriae by preventing CsgA aggregation in the periplasmic space [16,20]. Therefore, multiple mechanisms have been developed to prevent CsgA aggregation before its release in the extracellular environment. Furthermore, to appropriately induce or suppress CsgD expression, a minimum of 15 transcriptional regulators control the expression of the *csg* genes and are regulated by changes in factors including temperature, osmotic pressure, and pH [21,22,23]. These transcriptional regulators include the following: nucleoid proteins, such as Fis, H-NS, and IHF; two-component systems (TCSs), such as BasS/BasR, BtsS/BtsR, CpxA/CpxR, EnvZ/OmpR, and RstB/RstA and other transcriptional regulatory factors, such as MlrA, Cra, CRP, Crl, and RcdA [12,23,24,25,26,27,28,29,30,31,32,33,34]. Recently, we identified multiple new transcription factors, including YhjC (PlaR) and YjgJ (RcdB), that are potentially involved in the regulation of *csgD* expression. Thus, *E.coli* cells appear to respond to various environmental changes and finely regulate the formation of curli fimbriae [35].

Despite considerable progress in terms of the recent identification of the function of *csgC*—a constituent gene of the *csg* operon—the complete mechanism of curli fimbriae formation requires elucidation. Aside from controlling curli protein expression, CsgD regulates the expression of at least 20 other genes [36]; these include genes of unknown function that were predicted to be associated with curli fimbriae formation. Among these proteins, YccT is induced by CsgD, which directly binds to the 35–58 base pairs located upstream of the yccT transcription start site [36]. When taking its amino acid sequence into consideration, it is plausible that YccT may have a signal peptide at the N-terminus and be transported to the periplasmic region. STY1099, a Salmonella YccT ortholog, is reportedly involved in nitrate reductase activity, oxidoreductase activity, cellular uptake, and stress response and it plays a role in redox homeostasis under peroxide stress conditions [37]. However, the role of YccT remains largely unknown. The purpose of the present study was to elucidate YccT function. Herein, we demonstrated that YccT inhibited curli formation. Additionally, we provided new insights into the mechanisms underlying biofilm formation in bacterial species containing CsgD and YccT and causing infectious diseases in humans such as *Shigella* spp. and *Salmonella*. These data might provide the basis for developing inhibitors against biofilm formation by pathological bacteria.

## 2. Results

### 2.1. Inhibition of Curli Formation by CsgD Overexpression and Identification of the Responsible Gene

**Figure 1:**
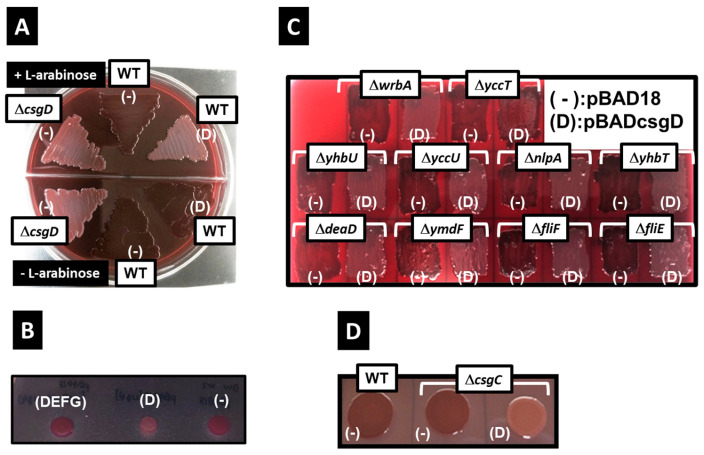
Inhibition of curli fimbriae formation via CsgD overexpression. Curli fimbriae formation was assessed through Congo red staining. (**A**) A *csgD* deletion strain (Δ*csgD*) transformed with the control vector pBAD18 (−) and wild-type (WT) strain transformed with pBAD18 (−) or pBADcsgD (D) were plated and incubated on a Congo red YESCA plate at 28 °C for 2 days in the presence (upper half) or absence (lower half) of 0.2% arabinose. (**B**) A wild-type strain (WT) transformed with pBADcsgDEFG (DEFG), pBADcsgD (D), or pBAD18 (−) were incubated on a Cong red YESCA plate at 28 °C for 2 days in the presence of 0.2% arabinose. (**C**) Effects of CsgD overexpression on curli fimbriae formation in strains deficient for CsgD target genes. Ten individual strains were generated by deleting CsgD targets (*wrbA*, *yccT*, *yhbU*, *yccU*, *nlpA*, *yhbT*, *deaD*, *ymdF*, *fliF,* and *fliE*). The control vector pBAD18 (−) or pBADcsgD (D) were introduced in each deletion strain. Cells were plated and incubated on a Congo red YESCA plate in the presence of 0.2% arabinose at 28 °C for 2 days. (**D**) Effect of CsgD overexpression on curli fimbriae formation in the CsgC deletion strain. A WT transformed with the control vector pBAD18 (−) and *csgD* deletion strains (Δ*csgD*) transformed with pBAD18 (−) or pBADcsgD (D) were plated and incubated on a Congo red YESCA plate in the presence of 0.2% arabinose at 28 °C for 2 days.

Strains without CsgD—a protein that activates the transcription of the *csgBAC* operon—lose their ability to form curli fimbriae and are not stained by Congo red [12], Figure 1A). In contrast, overexpression of *csgDEFG* induces *csgBAC* expression [38]. Indeed, we confirmed that the wild-type *E. coli* strain overexpressing *csgDEFG* derived from plasmid DNA induced curli formation to stain red, whereas the appearance of the CsgD-deletion strain was confirmed as a white colony on the Congo red plate (Figure 1B). Even when only CsgD is induced, *csgBAC* expression is significantly induced; however, the *csgDEFG* expression is induced only about 1.5 times higher than that of the control strain transformed with the empty vector [36]. For that reason, in CsgD overexpression from a multicopy plasmid, the expression of the CsgA monomer is induced; however, the transport proteins (CsgE/CsgF/CsgG) for exporting the CsgA monomer from the *E. coli* cell are hardly induced from the genome DNA [39]. Indeed, we confirmed that the colony of wild-type *E. coli* strain (BW25113) overexpressing CsgD is hardly stained by Congo red, thereby indicating the inhibition of the curli formation (Figure 1A,B). Additionally, a previous study reported that CsgD overexpression promoted cellulose synthesis and prevented colony staining by Congo red [40].

The BW25113 strain is useful for elucidating gene function as it can use an all-single-gene knockout mutant collection. However, the *E. coli* BW25113 strain used in this study did not produce cellulose fibers owing to the presence of a nonsense mutation at the sixth leucine residue of *bcsQ*, a gene of the cellulose synthesis operon, leading to a premature stop codon [38]. Therefore, the decreased Congo red staining induced by CsgD overexpression does not depend on the induction of cellulose synthesis. Previously, we were successful in identifying multiple novel target genes of CsgD [36]. Since these newly identified genes may comprise genes inhibiting curli fimbriae formation, the same experiment was performed using strains lacking one CsgD target gene (*deaD*, *fliF*, *fliE*, *nlpA*, *wrbA*, *yccT*, *yccU*, *yhbT*, *yhbU*, and *ymdF*). Curli fimbriae formation inhibition was prevented only in the *yccT* deletion strain (Figure 1B), suggesting that YccT was involved in the inhibition of curli fimbriae formation via CsgD overproduction. CsgC is the downstream gene of the *csgBAC* operon. Owing to the fact that CsgC inhibits CsgA polymerization [16], we determined whether CsgD-induced inhibition of curli fimbriae formation was mediated by CsgC. Curli fimbriae formation in the *csgC* deletion strain was remarkably inhibited through CsgD overexpression, which was similar to what was observed in the wild-type strain. This indicated that CsgD-induced inhibition was CsgC-independent (Figure 1D).

### 2.2. Effect of YccT Overexpression on Curli Fimbriae Formation

**Figure 2:**
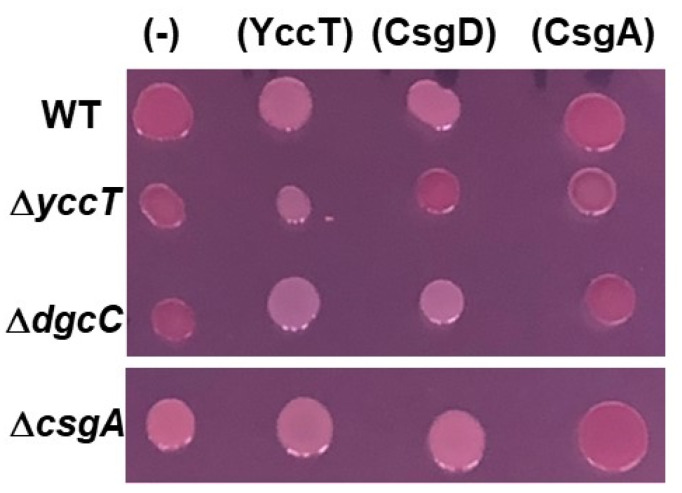
Inhibition of curli fimbriae formation through YccT overexpression. Curli fimbriae formation was tested by Congo red staining. Each strain (wild-type: WT or with a *yccT* [Δ*yccT*], *dgcC* [Δ*dgcC*], or *csgA* [Δ*csgA*] deletion) were transformed with the control vector pBAD18 (−), pBADyccT (YccT), pBADcsgD (CsgD), or pBADcsgA (CsgA). Cells were spotted and incubated on a Congo red YESCA plate in the presence of 0.2% arabinose at 28 °C for 2 days.

To confirm the effects of YccT on curli fimbriae formation, we constructed a YccT overexpression plasmid, pBADyccT. The effects of YccT overexpression on curli fimbria formation were assessed using Congo red plates in WT *E. coli* BW25113, *csgA* or *yccT* deletion mutants, and in *E. coli* with a deletion of *dgcC*, which encodes the c-di-GMP synthase involved in the regulation of cellulose synthesis by Bcs proteins under the control of CsgD. Owing to the fact that the *E. coli* BW25113 strain is unable to produce cellulose, the red colony color on the Congo red plates was considered to depend purely on curli fimbriae formation. The colonies of the *csgA* deletion strain were white, whereas the WT and *dgcC* deletion strains formed red colonies (Figure 2). All colonies, except those formed by the *yccT* deletion mutant, were white after CsgD overexpression (Figure 2, strains transformed with pBADcsgD). All strains overexpressing CsgA formed red colonies (Figure 2, strains transformed with pBADcsgA). Additionally, all strains overexpressing YccT formed white colonies (Figure 2, strains transformed with pBADyccT). The expression of all three genes was positively regulated by CsgD, which suggested that YccT was associated with the inhibition of curli fimbriae formation induced by CsgD overexpression.

### 2.3. Effects of CsgD Overexpression on csgA and yccT Expression

**Figure 3:**
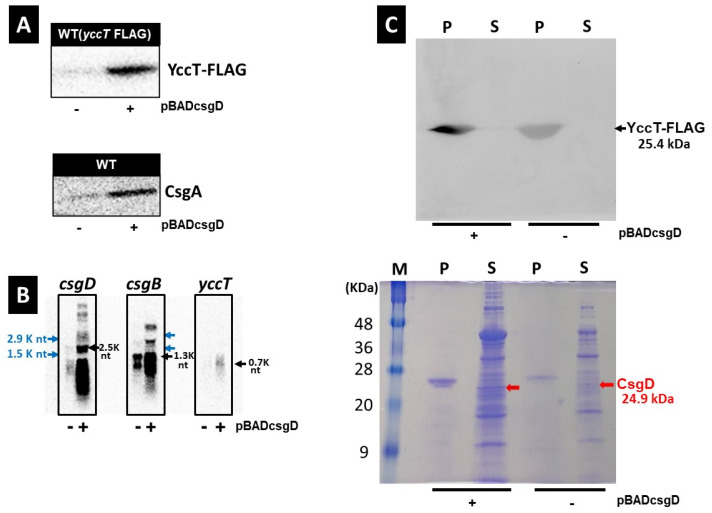
Effects of high CsgD expression on curli synthesis genes and YccT expression. (**A**) YccT-FLAG (upper panel) and CsgA (lower panel) expression in the BWWYTF(BW25113 YccT-FLAG) strain containing the control pBAD18 (−) or pBADcsgD (CsgD) vector. CsgA detection involved pretreating the samples with 90% formic acid (FA) and dried before resuspension in sample buffer. Anti-FLAG tag and anti-CsgA peptide antibodies were used to detect YccT-FLAG and CsgA via Western blot. (**B**) The expression of *csgD* (left panel), *csgB* (middle panel), and *yccT* (right panel) mRNAs in the BWWYTF(BW25113 YccT-FLAG) strain transformed with the control vector pBAD18 (−) or pBADcsgD (CsgD) is detected through Northern blot. (**C**) BWWYTF(BW25113 YccT-FLAG) strains containing the control vector pBAD18 (−) or pBADcsgD (CsgD) is shake cultured at 28 °C for 16 h in YESCA medium in the presence of L-arabinose (final concentration 0.02%). The periplasmic (P) and spheroplast (S) fractions are separated via ultracentrifugation after lysozyme processing. YccT-FLAG expression is detected by Western blot (upper panel) using an anti-FLAG tag antibody. The individual fractions are analyzed by Coomassie staining after SDS–PAGE (lower panel).

*csgBAC* expression is controlled by one promoter (*csgB*p) and its transcription is activated in a CsgD-dependent manner [12,36,41]. Similarly, the activation of *yccT* gene expression depends on CsgD [36]. Therefore, we investigated the mRNA and protein expression levels of CsgA, a major curli subunit, and YccT in cells overexpressing CsgD. To detect the YccT protein, a nucleotide sequence encoding a FLAG tag was inserted in frame before the stop codon of the *yccT* open reading frame (ORF) in the *E. coli* strain leading to the expression of a FLAG tag at the C-terminal ending of YccT. The resulting BWyccT-FLAG strain was prepared and grown for 12 h in preculture and 16 h in main culture (yeast extract casamino acids [YESCA] medium, final concentration of L-arabinose 0.02%). Western blot analyses were subsequently performed using an anti-FLAG antibody. YccT and CsgA protein expression was induced by CsgD overexpression (Figure 3A). Furthermore, the analysis of *csgD*, *csgB*, and *yccT* mRNA levels via Northern blot indicated that the expression of *csgD* and *csgB* were remarkably induced and *yccT* was moderately increased through CsgD overexpression, indicating a CsgD-dependent activation of their transcription (Figure 3B). Notably, although CsgD overexpression induces the expression of the curli component proteins CsgA and CsgB, it inhibited curli fimbriae formation (Figure 1 and Figure 2). These results suggested that YccT affected CsgA after CsgA transport in the extracellular space. The YccT sequence contains a signal peptide comprising 20 amino acids, suggesting that it is transported to and functions in the periplasmic space. Therefore, we investigated YccT expression in the periplasmic space after CsgD overexpression by Western blot analyses of BWyccT-FLAG strains containing the control (pBAD18) or csgD overexpressing (pBADcsgD) vector. YccT-FLAG was detected in the periplasmic fraction, but not in the spheroplast fraction, in both strains (Figure 3C). These data indicated that YccT, like CsgA, secretes into the periplasmic space.

### 2.4. Effect of YccT on CsgA Amyloid Fiber Formation In Vitro

**Figure 4:**
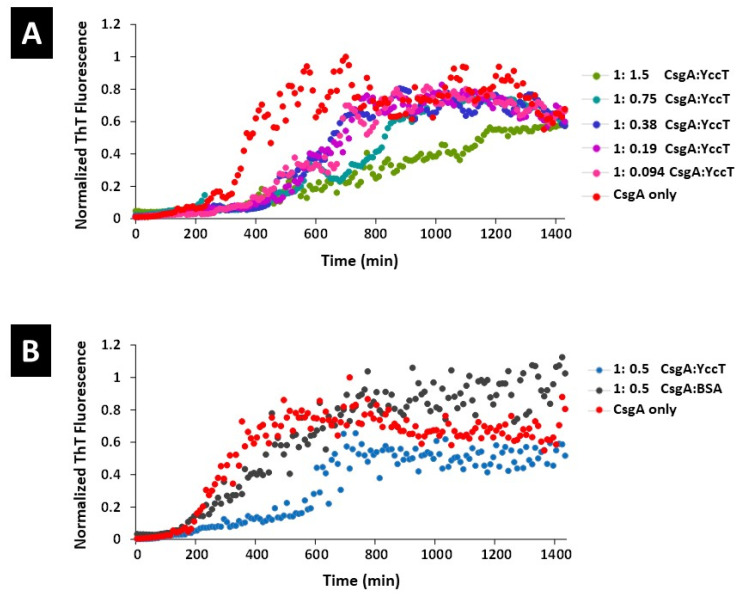
Detection of CsgA amyloid formation by ThT assay. (**A**) Samples of purified CsgA only or mixture of purified CsgA and YccT (ratios CsgA/YccT of 1.5, 0.75, 0.38, 0.19, or 0.094) were incubated at room temperature. The fluorescence intensity was determined every 10 min using the formula indicated in the text and was plotted on a graph. (**B**) Samples of purified CsgA only, purified CsgA (same volume) and YccT (0.5 times the amount of CsgA), or purified CsgA and BSA (0.5 times the amount of CsgA) as control were analyzed as described in (**A**). Each graph is representative of two independent experiments.

To determine whether YccT affected CsgA in the periplasmic region and to elucidate the underlying mechanism, the effects of purified YccT on CsgA fiber formation were examined in vitro using the thioflavin-T (ThT) assay. CsgA amyloid fiber formation involved a nucleation (3–4 h of gradual increase in the fluorescence intensity) and an elongation (4–10 h of a remarkable increase in fluorescence intensity) reaction. The maximal fluorescence intensity was reached after 10 h (Figure 4A,B). The addition of YccT—YccT/CsgA ratio of approximately 1/10 (0.094:1)—prolonged the nucleation by 6–7 h, and the inhibition of CsgA aggregate formation in the elongation phase was dependent on YccT concentration (Figure 4A). These results suggested that YccT interacted directly with CsgA and contributed to the inhibition of CsgA polymerization, thereby promoting its degradation, or inhibiting its transport from the periplasmic space to the extracellular space.

### 2.5. Effect of YccT Overexpression on CsgA Expression

**Figure 5:**
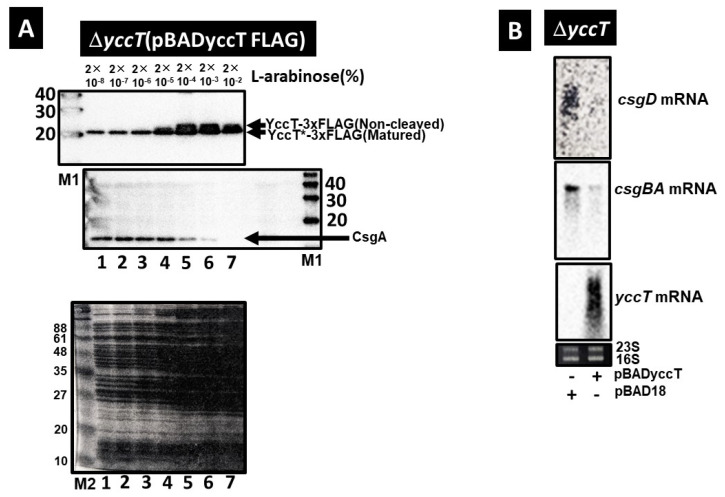
Effects of YccT overexpression on the expression of curli synthesis genes. (**A**) Full-length YccT-FLAG (non-cleaved), YccT*-FLAG (mature YccT-FLAG with cleaved signal peptide), and CsgA expression in the BWDyccT strain (with *yccT* deletion) transformed with pBADyccT-FLAG in the presence of several concentrations of L-arabinose (from 2 × 10^−9^ to 2 × 10^−3^%). Anti-FLAG tag and anti-CsgA peptide antibodies were used for the detection of YccT-FLAG and CsgA by Western blot. SDS PAGE was carried out for comparison of total proteins in the cells among each strain (lower panel). (**B**) The expression of *csgD* (upper panel), *csgB* (middle panel), and yccT (lower panel) mRNAs in the BWDyccT strain transformed with the control vector pBAD18 (−) or pBADyccT (YccT) in the presence of 2 × 10^−2^% L-arabinose was detected via Northern blot. All of *csgD*, *csgB* and *yccT* mRNA were detected with DIG-labelled *csgD*, *csgB* or *yccT* 3′ probes (see Section 4). The 23S and 16S rRNA were detected by ethidium bromide staining.

To clarify the effects of YccT overexpression on CsgA expression, we introduced the pBADyccT-FLAG vector into the yccT deletion strain to induce YccT-FLAG fusion protein expression. YccT*-FLAG (mature YccT-FLAG with the signal peptide cleaved by transport to the periplasmic region) was expressed for all arabinose concentrations of >2.0 × 10^−8^% (Figure 5A, upper panel). Moreover, YccT-FLAG is not transported to the periplasmic region, and it accumulated intracellularly when arabinose was added in amounts greater than 2.0 × 10^−4^% (Figure 5A, upper panel). In contrast, treatment with 2.0 × 10^−4^% L-arabinose induced a decrease in CsgA expression and almost no CsgA expression was detected when the arabinose concentration was 2.0 × 10^−2^% (Figure 5A). The effects on *csgD* and *csgBA* transcription of YccT overexpression were confirmed via Northern blot. Indeed, pBADyccT was introduced in the *yccT* deletion strain, and the expression of *csgBA* and *csgD* in this strain was significantly inhibited compared with that in the strain containing the control vector (Figure 5B). These results indicated that the repression of *csgBA* and *csgD* expression owing to YccT overexpression occurred at the transcriptional level.

### 2.6. Effects of Deletion of Signal Peptide Sequence of YccT on Curli Formation and YccT Localization

**Figure 6:**
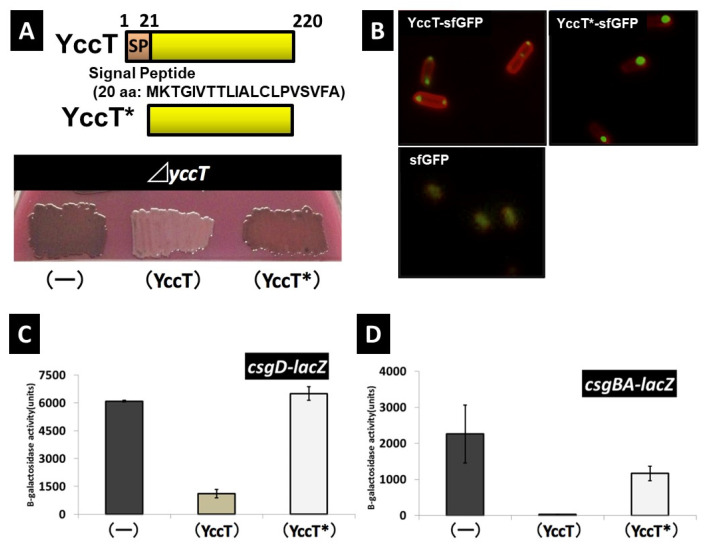
Effects of the deletion of YccT signal peptide on the curli fimbriae formation, YccT localization, and the expression of curli genes. (**A**) Curli fimbriae formation was assessed by Congo red staining. YccT* has a deletion of the signal peptide (first 20 N-terminal amino acids) (upper figure). The *yccT* deletion strain (Δ*yccT*) was transformed with the control pBAD18 (−), pBADyccT (YccT), or pBADyccT* (YccT*) vector. Cells were plated and incubated on a Congo red YESCA plate in the presence of arabinose (final concentration 0.2%) at 28 °C for 2 days. (**B**) Localization of YccT-sfGFP and YccT*-sfGFP in the wild-type strain (WT). The expression of sfGFP was used as negative control. The membranes of the *E.coli* cells were stained with FM4-64 (red). (**C**) The *csgD* promoter activity was determined by ß-galactosidase activity assay using a single copy *csgD* promoter-*lacZ* fusion (*csgD-lacZ*) in the WT containing the control pBAD18 (−), pBADyccT (YccT), or pBADyccT* (YccT*) vector. The cells were cultured in the presence of arabinose (final concentration 0.2%) at 28 °C for 16 h. (**D**) The *csgB* promoter activity was determined by ß-galactosidase activity using a single copy *csgB* promoter-*lacZ* fusion (*csgBA-lacZ*) in the WT containing the control pBAD18 (−), pBADyccT (YccT), or pBADyccT* (YccT*) vector. The cells were cultured in the presence of arabinose (final concentration 0.2%) at 28 °C for 16 h. The activity values represent the means ± SEs of triplicate experiments.

To confirm the effects of the deletion of the YccT signal peptide sequence on the repression of curli fimbriae formation induced by YccT overexpression, the yccT deletion strain was transformed with the plasmid containing YccT*. The color of colonies formed by bacteria transformed with the yccT or yccT* expression plasmid (pBADyccT or pBADyccT*, respectively) was investigated using the YESCA agar medium containing Congo red. The strain containing pBADyccT formed white colonies, whereas the strain transformed with pBADyccT* formed red colonies as did bacteria expressing the control vector (pBAD18) (Figure 6A). These results suggested that the transport of YccT to the periplasmic region plays an important role in repressing curli fimbria formation (Figure 6A).

The YccT-sfGFP and YccT*-sfGFP vectors in which sfGFP was subsequently fused with YccT and YccT*, respectively, were expressed in the BW25113 strain to investigate the localization of YccT in *E. coli* cells. Unipolar or bipolar distribution of the YccT-sfGFP signal was observed (Figure 6B). Since YccT was transported to the periplasm (Figure 3C), this distribution indicated that a part of YccT-sfGFP that was intended for transport to the periplasm remained in the cells. In contrast, most of the YccT*-sfGFP fluorescence signal followed a unipolar pattern and formed spots larger than those observed for YccT-sfGFP. The transport of YccT*-sfGFP to the periplasm space was expected to be blocked; as a result, YccT*-sfGFP remained unipolar owing to aggregation or interaction with some cell factors. Therefore, YccT might localize to the cell’s pole by interacting with unknown factors to transport it to the periplasmic region. Additionally, YccT without a signal peptide formed remarkable aggregates in cells and its localization was different, which may have been the main etiology for the loss of the inhibitory effect on curli fimbriae formation.

Next, the transcription of *csgBA* and *csgD* after YccT* overexpression was investigated to clarify the effects of the deletion of the YccT signal peptide on the expression of *csgBA* and *csgD*. The examination of each promoter activity using lacZ reporter strains revealed that deleting the YccT signal peptide restored *csgD* expression to the same level as that in the WT strain expressing the control vector. Similarly, *csgBA* expression was recovered to about 50% of that of the WT strain containing the control vector (Figure 6C,D). However, because *csgB* promoter activity was not recovered by approximately 50%, YccT*-sfGFP—which remains in cells—may be directly or indirectly involved in CsgD-dependent *csgB* induction. These results suggested that the signal peptide contributed to the bipolar or unipolar localization of YccT cells and its transport to the periplasm was directly or indirectly, through its interaction with unknown factors, involved in *csgD* and *csgBA* transcriptional inhibition.

### 2.7. Involvement of the Main Stress Response Systems in the Regulation of csgD and csgBA Expression by YccT

**Figure 7:**
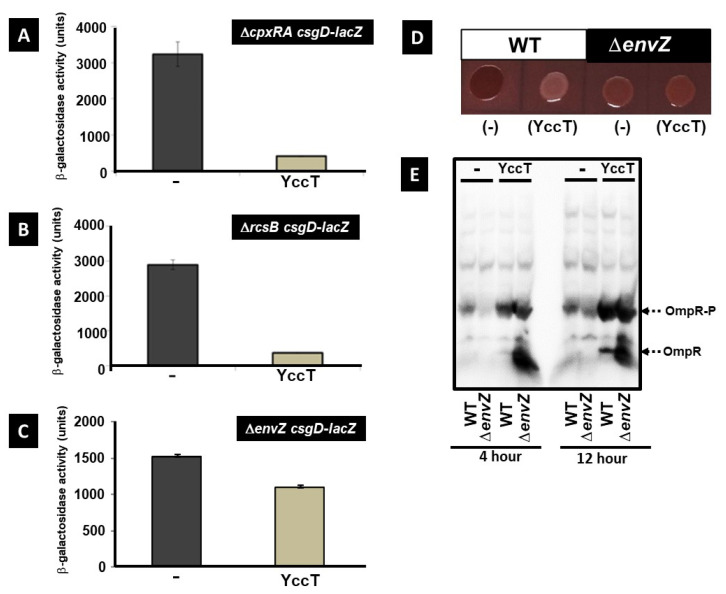
Effects of YccT overexpression on *csgD* expression in mutants showing deletion of the sensor and regulator from the two-component system related to the *csgD* expression. (**A**) The *csgD* promoter activity was determined through ß-galactosidase activity using a single copy *csgD* promoter-*lacZ* fusion (*csgD-lacZ*) in a *cpxRA* deletion strain (Δ*cpxRA*) transformed with the control pBAD18 (−) or pBADyccT (YccT) vector. The cells were cultured in the presence of arabinose (final concentration 0.2%) at 28 °C for 16 h. The activity values represented the means ± SE of triplicate experiments. (**B**) The *csgD* promoter activity was determined by ß-galactosidase activity assay using a single copy *csgD* promoter-*lacZ* fusion (*csgD-lacZ*) in *rcsB* deletion strain (ΔrcsB) transformed with the control pBAD18 (−) or pBADyccT (YccT) vector. The cells were cultured in the presence of arabinose (final concentration 0.2%) at 28 °C for 16 h. The activity values represent the means ± SEs of triplicate experiments. (**C**) The *csgD* promoter activity was determined using the ß-galactosidase activity assay using a single copy *csgD* promoter-*lacZ* fusion (*csgD-lacZ*) in *envZ* deletion strain (Δ*envZ*) transformed with the control pBAD18 (−) or pBADyccT (YccT) vector. The cells were cultured in the presence of arabinose (final concentration 0.2%) at 28 °C for 16 h. The activity values represent the means ± SEs of triplicate experiments. (**D**) Effect of *envZ* deletion on the inhibition of curli fimbriae formation induced by YccT overexpression. Curli fimbriae formation was detected by Congo red staining. Wild-type (WT) or envZ deletion (Δ*envZ*) strains containing the control pBAD18 (−) or pBADyccT (YccT) vector were incubated on a Congo red YESCA plate in the presence of 0.2% arabinose at 28 °C for 2 days. (**E**) Effects of YccT overexpression on the phosphorylation state of intracellular OmpR. Phosphorylated OmpR (OmpR-P) was detected using a Phos-tag SDS–PAGE assay. WT or envZ deletion (Δ*envZ*) strains containing the control pBAD18 (−) or pBADyccT (YccT) vector were grown in YESCA medium in the presence of 0.2% arabinose at 28 °C for 4 or 12 h. Purified His-tagged OmpR was used for detecting the position of phosphorylated or nonphosphorylated OmpR.

Numerous transcription factors have been involved in the regulation of *csgD* expression [35,42]. In particular, the signal transduction systems CpxA/R, RcsBAD, and EnvZ/OmpR, which are involved in the stress response, play a major role in regulating *csgD* expression [23,43,44]. To determine the involvement of these stress response systems in the repression of *csgD* expression induced by YccT overexpression, *csgD* expression in *cpxAR*, *rcsB*, and *envZ* deletion strains was investigated. YccT overexpression in the *cpxRA* and *rcsB* deletion strains induced a significant reduction in *csgD* expression compared with control strains (Figure 7A,B). However, this decrease was similar to that observed in the WT strain, suggesting that the Cpx and Rcs systems were not involved in the mechanism of *csgD* expression repression induced by YccT overexpression. In contrast, in the *envZ* deletion strain, the decrease in *CsgD* expression by YccT overexpression (~25%) was less compared to that of the WT strain (Figure 6C and Figure 7C) or the strain expressing the control vector (Figure 7C). The colonies of the WT strain containing the control vector were red on Congo red plates, whereas those of the *envZ*-deficient strain were pale red (Figure 7D, first and third pictures, respectively). In contrast, the WT strain overexpressing YccT formed white colonies, whereas the pale red color of the *envZ* deletion strain was maintained after YccT overexpression (Figure 7D, second and fourth pictures, respectively).

Next, we performed a Phos-tag sodium dodecyl sulfate–polyacrylamide electrophoresis (SDS–PAGE) assay to assess the effects of YccT overexpression on the phosphorylation state of intracellular OmpR. The amount of phosphorylated OmpR was greater in the YccT overexpressing WT and *envZ* deletion strains (Figure 7E). Additionally, an increase in non-phosphorylated OmpR was observed in the *envZ* deletion strain, which was attributable to *envZ* deletion. OmpR phosphorylation in the stationary phase is thought partially independent of EnvZ, and OmpR in *envZ* deletion strains is phosphorylated using other phosphate group donors such as acetyl phosphate [21,45]. Transcriptome analysis of YccT overexpressing cells revealed significant repression of *ompF* expression, suggesting that the stabilization of intracellular phosphorylated OmpR by YccT overproduction caused the repression of *csgD* expression.

### 2.8. Effect of EnvZ/OmpR on YccT Localization

**Figure 8:**
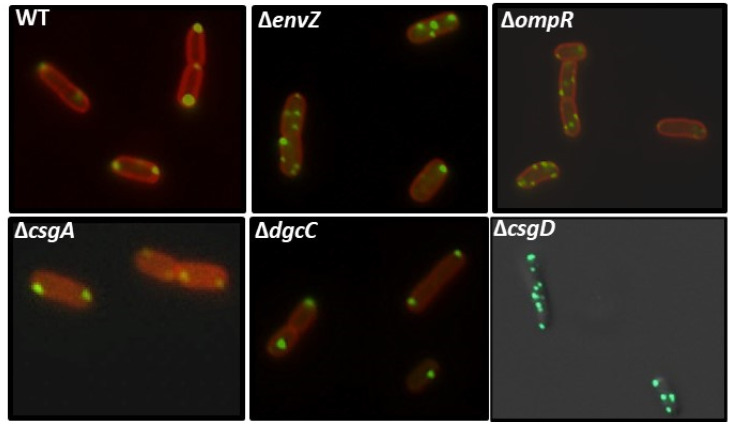
Curli fimbriae formation and YccT localization in various deletion mutants. Localization of YccT-sfGFP in each deletion strain. The wild-type (WT) strain or strains with a deletion of *envZ* (Δ*envZ*), *ompR* (Δ*ompR*), *csgA* (Δ*csgA*), *dgcC* (Δ*dgcC*), or *csgD* (Δ*csgD*) were transformed with pBADyccT-sfGFP and grown in LB medium in the presence of 0.02% (final concentration) arabinose at 37 °C for 2 h. The membranes of the *E.coli* cells (except that of the Δ*csgD* mutant) were stained with FM4-64.

Owing to the fact that the amount of OmpR in cells increased after YccT overexpression, YccT might be involved in activating OmpR expression or stabilizing OmpR. Therefore, we investigated YccT-sfGFP localization in *E. coli* strains lacking *envZ* or *ompR*. In the WT strain, YccT-sfGFP was localized at both poles of the cells, whereas in the *envZ* and *ompR* deficient strains, multiple fluorescent spots were observed in most cells (Figure 8). Additionally, the analysis of the fluorescent spots in the *ompR* deletion strain by deconvolution revealed that bright spots were present along the cell membrane (Figure S1). These results suggested that the deletion of *ompR* and *envZ* facilitated the formation of YccT-sfGFP aggregates and OmpR/EnvZ was involved in YccT localization to the cell poles. In contrast, the localization of YccT-sfGFP was not affected in the *csgA*-deficient strain, suggesting that CsgA was not involved in the localization of YccT to the cellular poles and YccT and CsgA did not interact in the cytoplasm.

## 3. Discussion

In the present study, we aimed to clarify the mechanisms through which the induction of CsgD expression inhibits curli fimbria formation and identified a CsgD-regulated periplasmic protein YccT as a potential inhibitor. Therefore, we propose to rename “YccT” to “CsgI” as the inhibitor of curli synthesis. We confirmed that the expression of curli subunits and YccT was induced by CsgD overexpression, suggesting that YccT inhibited the aggregation or extracellular transport of CsgA in the periplasmic region. Previously, a model in which CsgC specifically inhibits CsgA polymerization in the periplasmic space and facilitates CsgA extracellular secretion has been proposed [20]. Although the functions of YccT and CsgC are equivalent, their amino acid sequences are significantly different. The structure and active sites of YccT are currently under investigation.

The present data suggest that YccT interacted with CsgA in the periplasmic space and was involved in the inhibition of CsgA extracellular transport and/or degradation. Additionally, CsgC and YccT were simultaneously induced via CsgD overexpression. However, since curli fimbriae formation was inhibited in the *csgC* deletion strain as in the WT strain, it was likely that CsgC mediated the inhibition of curli fimbriae formation in CsgD overexpression conditions, suggesting that YccT and CsgC function independently of each other in the periplasmic space. In the early stage of biofilm formation in *E. coli,* CsgD is induced by multiple transcription factors such as IHF, which is a nucleoid protein, and OmpR, a transcription factor, and activates curli fimbriae (constituent subunits CsgA and CsgB) formation [21,23,29]. Our data suggested that YccT was simultaneously induced with curli fimbriae and interacted with the curli subunit CsgA in the periplasmic space to participate in CsgA degradation and reduce the excessive accumulation of curli constituting subunits in the periplasmic region. In the future, the interaction between CsgA and YccT in the cell and periplasmic space will be further investigated. Furthermore, we demonstrated that *csgD* expression was repressed when YccT was overexpressed. In YccT* (YccT without signal peptide), *csgBA* and *csgD* expression was restored and curli fimbriae formation occurred. When periplasmic proteins are overexpressed and aggregate in the periplasmic space, *csgD* expression is predicted to repress and envelope stress response systems, such as the Cpx and Rcs systems, thereby inducing the expression of genes encoding proteases and molecular chaperones are activated [25,43,44,46,47]. However, in this study, the involvement of Cpx and Rcs in the repression of *csgD* expression induced by YccT overexpression was not confirmed; however, the repression of *csgD* expression was lost in the *envZ* deletion strain transfected with the YccT overexpression plasmid. Therefore, the repression of *csgD* expression by YccT might be induced through the two-component regulatory system EnvZ/OmpR.

EnvZ regulates the expression of many target genes including genes encoding outer membrane proteins such as OmpF and OmpC by controlling the phosphorylation state of the paired cytoplasmic response regulator OmpR in response to changes in external osmolality. OmpC expression is preferentially induced via high osmotic conditions, whereas OmpF expression is induced by low osmotic conditions [48,49,50]. The EnvZ/OmpR system is activated not only by osmotic pressure changes but also by low pH and oligotrophic conditions [51,52,53]. Previous transcriptome analysis has shown an altered expression of more than 100 genes in *ompR/envZ* deletion mutants [54]. Additionally, the inner membrane protein MzrA modulates EnvZ and affects OmpR phosphorylation levels [55]. The detailed molecular mechanisms underlying YccT-associated inhibition of curli fimbriae formation remain unclear. In particular, it is necessary to investigate the interaction of YccT with EnvZ and OmpR and its relationship with the products of genes controlled by the EnvZ/OmpR system. In the present study, YccT overexpression induced an increase in OmpR levels and OmpR was more phosphorylated in the WT and *envZ* deletion strains overexpressing YccT, whereas the levels of OmpR dephosphorylation were higher in the *envZ* deletion strain. Therefore, YccT is likely stimulating the expression or stabilization of OmpR. OmpR phosphorylation depends on its cognate sensor kinase EnvZ and phosphate donors such as acetyl phosphate and carbamoyl phosphate [56,57,58,59]. The mechanism of YccT-induced OmpR phosphorylation is unknown; however, YccT might contribute to OmpR phosphorylation by activating EnvZ in the periplasmic space or by stabilizing OmpR. The promoters of *ompC* and *ompF,* which are regulated by EnvZ/OmpR, contain binding sites (F1, F2, F3, and F4 for *ompF* and C1, C2, and C3 for *ompC*) for phosphorylated OmpR. The hierarchical binding of phosphorylated OmpR to the F1 and F2 sites or F1, F2, and F3 sites in the *ompF* promoter activates *ompF* transcription, whereas higher levels of intracellular phosphorylated OmpR bind to the F4 site and repress the transcription of *ompF* [60,61,62]. In *Salmonella*, phosphorylated OmpR binds the *csgD* promoter at the D1, D2, and D3–D6 sites. OmpR binding to D1 is important for activating *csgD* expression, whereas its further binding to D2 represses *csgD* expression in vivo [21]. In contrast, in *E.coli*, only the D1 site was observed with the homologous region of the *csgD* promoter region [22,23], whereas the recent Genomic SELEX study using phosphorylated OmpR has shown the OmpR binding site is located downstream of *csgD*_P1_. [63].

Thus, the elevated phosphorylated OmpR levels induced by YccT overexpression might promote phosphorylated OmpR binding downstream of *csgD*_P1_ of the *csgD* promoter and repress csgD expression. In the *envZ* deletion strain, YccT overexpression also induced substantial levels of dephosphorylated OmpR, suggesting that the inhibitory effect on *csgD* expression was prevented by the competition of dephosphorylated OmpR with phosphorylated OmpR. The mechanisms by which YccT promotes OmpR expression or stabilization are currently under investigation. In addition, the genomic SELEX study has shown that the *yccT* promoter is a target of OmpR; however, the mechanisms of *yccT* expression regulation by OmpR remain elusive [63].

Microscopic analyses of YccT localization in *E. coli* cells revealed that YccT-sfGFP foci were found at one of both cellular poles in the WT strain. In contrast, the signal peptide deletion mutant (YccT*-sfGFP) formed aggregates in one cellular pole. Recently, the bipolar localization of *Salmonella enterica* YccT (homology with *E. coli* YccT: 71%) has been shown [37]. Our results in *E.coli* cells are partially in agreement with these data, suggesting that similar mechanisms are involved in YccT localization in *E.coli* and *Salmonella*. Additionally, coimmunoprecipitation assays showed that *Salmonella enterica* YccT may interact with MreB, DnaK, or OmpR [37]. MreB and the molecular chaperone DnaK are involved in localizing several response regulators to the poles of cells [64]. Moreover, OmpR-GFP foci have a bipolar distribution [64,65]. Analyses using OmpR-YFP have shown that EnvZ-dependent phosphorylated OmpR-YFP foci are found near the poles in the presence of the OmpR target gene promoter on plasmid DNA [66]. Thus, the distribution pattern of response regulators might be closely related to their function as transcription factors [64].

In *ompR* or *envZ* deletion strains, YccT-sfGFP formed many foci and presented a spiral localization pattern along the membrane. Additionally, the localization of YccT-sfGFP did not change in the *csgA* deletion strain suggesting that CsgA does not interact with intracellular YccT and does not affect YccT localization. However, in the *csgD* deletion strain, YccT-sfGFP formed many foci in the cells comparable to those observed in the *ompR* or *envZ* deletion strain. These data suggested that the factors involved in YccT localization to the poles and transport to the periplasm are regulated by CsgD. Since OmpR/EnvZ is essential for *csgD* expression, the changes in YccT-sfGFP localization in *ompR or envZ* deletion strains might be due to the effects on CsgD expression (Figure 8). The strains without major CsgD-controlled genes csgA and dgcC showed YccT-sfGFP localization similar to that of the wild-type strain, suggesting the involvement of other CsgD-controlled genes in the localization.

**Figure 9:**
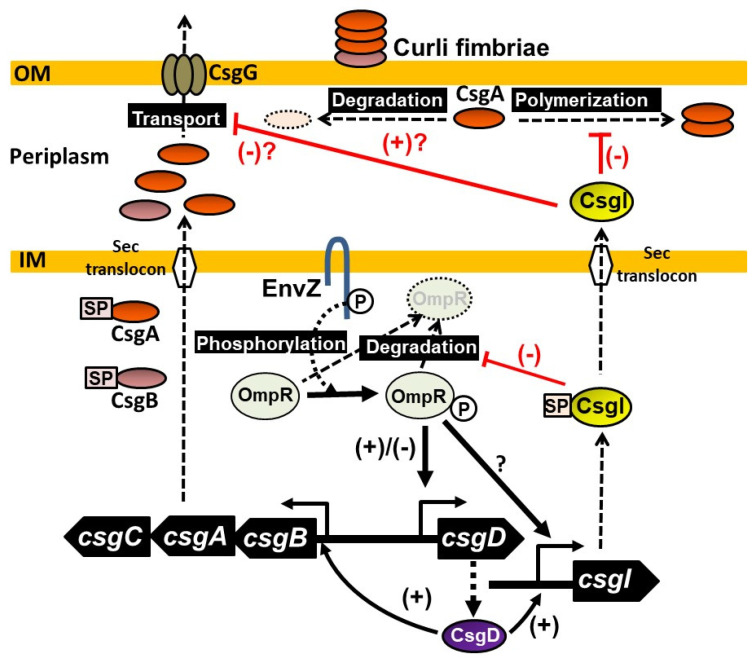
Schematic model of curli synthesis mechanisms and YccT inhibitory action.

The molecular chaperone DnaK is involved in the transport of stable CsgA into the periplasmic space and the stabilization of CsgD and plays an important role in curli fimbria formation [67,68]. Because the expression of YccT was induced in a CsgD-dependent manner, DnaK might be important for YccT localization. It is also possible that the inhibition of curli fimbria formation induced by YccT overexpression results from the competition of YccT with CsgA for DnaK-mediated transport into the periplasmic space. Based on these results, we propose a model of the mechanisms triggered by YccT to inhibit curli fimbriae formation (Figure 9). Interestingly, in *Salmonella*, FitT, which prevents flagellar protein aggregation and contributes to extracellular transport, plays a role in fine-tuning the expression of the flagellar gene [69]. Therefore, YccT might act similarly to FliT.

In conclusion, our data demonstrate that YccT is a novel inhibitor of curli formation by inhibiting both CsgA polymerization and curli gene expression. Thus, we propose to rename YccT CsgI as an inhibitor of curli synthesis.

## 4. Materials and Methods

### 4.1. Bacterial Strains and Culture Conditions

*E. coli* BW25113 and the gene deletion strains used in this study are listed in Table 1. These strains were grown in YESCA medium at 28 °C or in lysogeny broth (LB) medium at 37 °C with constant shaking at 140 rpm. *E. coli* BL21(DE3) strain was used for the expression and purification of YccT without signal peptide and CsgA. The one-step recombination system and PCR products were used to add the FLAG tag sequence to the YccT gene in the *E.coli* genome [70]. For the reporter assay to detect *csgD* promoter activity, a single copy promoter-*lacZ* fusion was generated in the genome of *E.coli* according to a method described previously [71]. All constructed strains are shown in Table 1.

### 4.2. Plasmid Construction

For the construction of His-tagged YccT or CsgA expression plasmids, DNA fragments containing the *yccT* or *csgA* ORFs were amplified by PCR using *E. coli* BW25113 genome DNA as template and a pair of specific primers (primer sequences are in Table 1). After digestion with *NotI* and *NdeI*, each PCR fragment was inserted into pET21(a) at the corresponding site. For the construction of an arabinose-inducible YccT or YccT-FLAG expression plasmid, a DNA fragment containing each gene’s ORF was amplified by PCR using *E. coli* BW25113 genome DNA as a template and a pair of gene-specific primers (see Table 1). After digestion with *Eco*RI and *Xba*I, the PCR-amplified fragment was inserted at the corresponding site of pBAD18 [75] to generate the plasmids pBADyccT and pBADyccT-FLAG. For the construction of the expression plasmids containing YccT-sfGFP or the mutant YccT*-sfGFP with a deletion of the signal peptide, a linearized DNA fragment of pBAD24 was prepared by PCR using Prime STAR Max (Takara) and fused with YccT-sfGFP or YccT*-sfGFP fragment using Infusion cloning kit (Takara) (for primer sequences see Table 1). The Big Dye Terminator Cycle Sequencing V 3.1 Kit (Applied Biosystems) was used for the sequence analysis of each plasmid.

### 4.3. Northern Blot Analysis

Overnight cultures were incubated in 30 mL YESCA medium at 28 °C for 16 h to prepare total RNA for Northern blot analysis. RNA purification and Northern blot analysis were performed as described previously [23]. DIG-labeled DNA fragments were amplified by PCR using BW25113 genomic DNA (50 ng) as a template, DIG-11-dUTP (Roche Basel, Switzerland) and dNTPs as substrates, gene-specific forward and reverse primers (csgD-N-F and csgD-N-R for the *csgD* probe; csgB-s-N and csgB-t-N for the *csgB* probe; yccT-s-N and yccT-t-N for the *yccT* probe; Table 1), and Ex-Taq DNA polymerase (Takara bio, Shiga, Japan). Briefly, 4 μg of total RNA was denatured in formaldehyde-MOPS gel loading buffer at 65 °C for 10 min, separated by electrophoresis on a 2% agarose gel containing formaldehyde, and transferred onto a nylon membrane (Roche). Hybridization with the DIG-labeled probe was performed overnight at 50 °C using the DIG easy Hyb system (Roche). To detect DIG-labeled probes, the membranes were treated with anti-DIG-AP Fab fragments and CDP-Star (Roche) and images were scanned with Typhoon Trio (cytiva, Marlborough, MA, USA).

### 4.4. Expression and Purification of YccT and CsgA

The expression and purification of His-tagged YccT were conducted following the standard procedure [76]. His-tagged transcription factors were expressed in E. coli BL21(DE3) transformed with YccT expression plasmid (pETyccT-His).

First, pETyccT and pETcsgA (Table 1) were introduced into BL21(DE3). The transformed bacteria were cultured in 500 mL LB medium containing ampicillin (100 µg/mL) to an OD_600_ of 0.9. Then, IPTG at a final concentration of 1 mM was added and the culture was incubated for 3 h shaking. Afterward, the cells were collected by centrifugation (5000 rpm for 20 min).

For YccT purification, 5 mL of Bugbuster (Takara bio) per 1 g of pellet, 10 µL of 0.1 M PMSF, 2 µL of DNase (5 U/µL), and 2 µL of RNase (10 mg/mL) were added to the pellet. The samples were rotated for 20 min and then centrifuged at 10,000 rpm for 20 min. The supernatants were mixed with 1 mL of Ni-NTA agarose (QIAGEN, Hilden, Germany) and shaken on a rotator for 1 h. The solutions were then transferred to a column (Muromachi Chemicals, Inc., Tokyo, Japan), washed with 10 mL lysis buffer (50 mM Tris/HCl pH 8.0 at 4 °C and 100 mM NaCl), and eluted with 2 mL lysis/imidazole buffer. A 2-mL sample was transferred to a PD-10 column (GE Healthcare, Chicago, IL, USA) equilibrated with Kpi buffer, and the sample drained with 3.5 mL Kpi buffer was used for the ThT assay.

For CsgA purification, the collected pellet was dissolved in 25 mL of 8 M guanidine hydrochloride (GdnHCl) and 50 mM K2HPO4 and stirred with a stirrer at 4 °C for 2 days. The mixture was centrifuged at 10,000 rpm for 20 min at 4 °C, the supernatant was collected, ultrasonically crushed (Astrason XL2020, MISONIX, Farmingdale, NY, USA), mixed with 1 mL of Ni-NTA agarose, and shaken on a rotator for 1 h. The solution was transferred to a column (Muromachi Chemicals, Inc.), washed with 10 mL of 50 mM Kpi buffer, and CsgA protein was eluted with 2 mL of 125 mM imidazole/Kpi buffer. The eluted sample was passed through an ultrafiltration filter (Millipore; Amicon Ultra-4 Centrifuged Filter Devices 30 KDa) by centrifugation at 7500× *g*. Then, the filtered solution was transferred to a PD-10 column equilibrated with Kpi buffer and the sample drained with 3.5 mL Kpi buffer was passed through an ultrafiltration filter (Millipore; Amicon Ultra-4 Centrifuged Filter Devices 10 KDa) by centrifugation at 7500× *g*. The solution in the concentrated column was measured and used for the ThT assay. The purity of YccT and CsgA used in this study was verified by SDS–PAGE.

### 4.5. Periplasmic Extract Preparation

The BWyccT-FLAG introduced with pBADcsgD or pBAD18 were cultured with shaking at 28 °C for 16 h in 10 mL of YESCA medium. These culture solutions were transferred into a 50 mL Falcon tube and collected by centrifugation (4 °C, 5000× *g*, 10 min). After removing the supernatant from each sample, 5 mL of Spheroplast buffer (30% sucrose, 5 mM EDTA-2Na, 10 mM Trisbase) was added to suspend, and 5 mg of lysozyme was added and mixed. After cooling on ice for 45 min, ultracentrifugation (4 °C, 26,000× *g*, 10 min, himac CS100GX) was performed, and the supernatant was used as a periplasmic fraction. The pellet was suspended by adding 5 mL of Spheroplast buffer, which was used as a spheroplast fraction. Each fraction was transferred to a size exclusion spin column (Vivaspin 6; GE Healthcare), centrifuged (room temperature, 10,000× *g*, 30 min) and concentrated. The periplasmic fraction and the spheroplast fraction were electrophoresed by SDS-PAGE, and YccT-FLAG was detected by the Western blot assay using a FLAG antibody.

### 4.6. In Vitro ThT Amyloid Polymerization Assay

The ThT assay was performed as described previously [77]. Purified CsgA and YccT samples and 20 µM ThT were added to a Nunc F96 MicroWell Black Polystyrene Plate (Thermo Fisher Scientific, Waltham, MA, USA). A negative control was prepared with BSA. The background was measured using a sample containing only Kpi buffer and ThT. The fluorescence intensity was measured using ARVO (Perkin Elmer, Waltham, MA, USA) (emission 495 nm, excitation 438 nm). To generate a graph, the fluorescence intensity was standardized using the following formula:

[Fi − F_0_]/[F_max_ − F_0_]

Fi = Fluorescence intensity of each sample
F_max_ = the highest fluorescence intensity in samples with CsgA
F_0_ = background fluorescence intensity obtained for samples containing only Kpi buffer and ThT

### 4.7. Determination of YccT Localization Using sfGFP

*E. coli* BW25113 strain transformed with an expression plasmid containing the sequence of YccT or YccT-sfGFP with or without signal peptide were shaken in LB medium supplemented with L-arabinose (final concentration: 0.02%) at 37 °C for 2 h. Then, 200 μL of 1% glutaraldehyde was added to 100 μL of culture solution, and the mixture was incubated at room temperature for 2.5 h. For the staining of the *E. coli* membrane, FM4-64 (final 1 μM) was added and then centrifuged at 9,100 g for 1 min, and pellets were re-suspended in 20 μL of PBS (-). Each sample was observed using a green or red filter of an optical fluorescence microscope (Axioimager M1; Carl Zeiss Meditec AG, Jena, Germany) with 630 times magnification. The automatic adjustment mode was used for the exposure time when taking a photomicrograph. The photomicrographs obtained with both filters were merged using ImageJ software (NIH).

### 4.8. Reporter Assay of the csgD Promoter

The *csgD*-*lacZ* and *csgB-lacZ* reporter strains used in this study are listed in Table 1. The measurement of ß-galactosidase activity was performed as described previously [78].

### 4.9. Western Blot

Overnight cultures of *E. coli* strains were inoculated into 10 mL YESCA medium and cultured for 16 h shaking at 28 °C. Then, the cultures were transferred to a 15-mL Falcon tube and centrifuged at 10,000 rpm for 10 min at 4 °C. The supernatant was discarded and 5 mL Bugbuster Protein Extraction Reagent (Novagen, Darmstadt, Germany), 1 µL rLysozyme™ solution (Merck, Darmstadt, Germany), and 1 µL of 100 mM PMSF were added to 1 g of pellet. The mixture was gently inverted and stirred on a rotator for 20 min. It was then centrifuged at 15,000 rpm for 20 min at 4 °C and the supernatant was transferred to a new 1.5-mL tube. To precipitate CsgA protein, 300 µL of the supernatant was mixed with 60 µL 1 M NaCl in a new 1.5-mL tube and left on ice for 10 min to precipitate CsgA. The mixture was then centrifuged at 20,000 rpm for 10 min at 4 °C and the supernatant was discarded. The pellet was resuspended in 30 µL of 90% formic acid and dried in a centrifugal concentrator (CC-105: Tomy Seiko, Tokyo, Japan) before being suspended in 15 µL of 8 M urea. After SDS–PAGE, proteins were transferred to a PVDF membrane using Trans-blot SD semi-dry transfer cell (Bio-Rad, Hercules, CA, USA). YccT-FLAG and CsgA were detected with primary antibodies recognizing the FLAG tag (Sigma-Aldrich, St. Louis, MO, USA) or CsgA peptide (Eurofins; gifted by Dr. Shinya Sugimoto) and anti-rabbit IgGs (H + L) (MP Biomedicals, LLC-Cappel Products, Santa Ana, CA, USA) as a secondary antibody.

### 4.10. Detection of OmpR Phosphorylation by Phos-Tag SDS–PAGE

Each plasmid (pBAD18, pBADyccT, or pBADdspyccT) was introduced into BW25113 or JW3367 cells, which were cultured in 10 mL LB medium containing ampicillin (100 µg/mL) to an OD_600_ of 0.9. Then, arabinose at a final concentration of 1 mM was added and the culture was incubated for 4 or 12 h shaking. Afterward, the cells were collected by centrifugation (5000 rpm for 20 min). Bug Buster (Takara) was added to the pellets, which were then stored at −80 °C for 1 day. The pellets were thawed on ice, and lysozyme solution (1 μL/1 mL Bugbuster) and 1 μL of 100 mM PMSF were added and mixed well. The mixture was incubated at room temperature for 20 min. After centrifugation at 15,000 rpm for 20 min at 4 °C, the cells were dissolved in 4 × SDS loading dye, and proteins were separated by electrophoresis on a Super Sep Phos-tag SDS–PAGE (Fujifilm-Wako, Osaka, Japan) containing 25 μM Phos-tag™ acrylamide and 50 μM MnCl2. The gel was shaken in 5 mM EDTA/Western buffer (Nacalai Tesque, Kyoto, Japan) three times 10 min. The gel was shaken for an additional 10 min in Western buffer and then transferred to a nitrocellulose membrane using a Bio-Rad semi-dry transfer device. Proteins were then detected following a standard Western blot protocol and using rabbit anti-OmpR primary antiserum (gifted by Akira Ishihama) and anti-rabbit IgGs (H + L) (MP Biomedicals, LLC-Cappel Products) as a secondary antibody. Bands were detected on LAS-1000 (Fuji Film, Tokyo, Japan) using an Immobilon western chemiluminescent HRP substrate (Millipore, Darmstadt, Germany). The position of the phosphorylated OmpR was determined using a molecular weight marker and purified His-tag OmpR treated with acetyl phosphate.

### 4.11. Congo Red Plate Assay to Detect the Curli Fimbriae Formation

The Congo red plate assay was performed as described previously [79]. *E. coli* strains were grown at 28 °C for 48 h on a YESCA plate containing 50 μg/mL Congo red and 10 μg/mL Coomassie blue.

## Figures and Tables

**Table 1:** Strains, plasmids, and primers used in this study.

Strain	Genotype	**Source or Reference**
DH5α	F−, 80dlacZΔM15, Δ(lacZYA-argF)U169, deoE, recA1, endA1 hsdR17(rK−,mK+), phoA, supE44, λ−, thi-1, gryA96, relA1	Takara bio, Shiga, Japan
BL21(DE3)	F−, lon-11, Δ(ompT-nfrA)885, Δ(galM-ybhJ)884, λDE3 [lacI, lacUV5-T7 gene 1, ind1, sam7, nin5], Δ46, [mal+]K-12(λS), hsdS10	[72]
BW25113	F−, Δ(araD-araB)567, ΔlacZ4787(::rrnB-3), λ− rph-1, Δ(rhaD-rhaB)568, hsdR514	[73]
BW27559	BW25113 Δ(cpxR)623	[54]
BW27870	BW25113 ΔrcsB1320	[54]
JW1023	BW25113ΔcsgD::Kan	[74]
JW0947	BW25113ΔyccT::Kan	[74]
JW0989	BW25113ΔwrbA::Kan	[74]
JW5130	BW25113ΔyccU::Kan	[74]
JW3127	BW25113ΔyhbU::Kan	[74]
JW3635	BW25113ΔnlpA::Kan	[74]
JW3126	BW25113ΔyhbT::Kan	[74]
JW5531	BW25113ΔdeaD::Kan	[74]
JW5136	BW25113ΔymdF::Kan	[74]
JW1922	BW25113ΔfliF::Kan	[74]
JW1921	BW25113ΔfliE::Kan	[74]
JW1026	BW25113ΔcsgC::Kan	[74]
JW3367	BW25113ΔenvZ::Kan	[74]
JW3368	BW25113ΔompR::Kan	[74]
JW5665	BW25113ΔbcsA::Kan	[74]
BWΔenvZ	Km resistance marker of JW3367 eliminated with pCP20	This study
BWyccT-FLAG	BW25113yccT::3×FLAG	This study
BWWcsgD-lacZ	BW25113 λcsgD-lacZ	[36]
BWWcsgBA-lacZ	BW25113 λcsgBA-lacZ	[36]
BWΔcpxRAcsgD-lacZ	BW27559 λcsgD-lacZ	This study
BWΔrcsBcsgD-lacZ	BW27870 λcsgD-lacZ	This study
BWΔenvZcsgD-lacZ	BWΔenvZ λcsgD-lacZ	This study
Plasmid	Genotype	Source or Reference
pBAD18	araC, rrnBT, amp	[75]
pBAD24	araC, rrnBT, amp	[75]
pBADcsgD	pBAD18 with fragment containing yccT ORF	This study
pBADyccT	pBAD18 with fragment containing yccT ORF	This study
pBADyccT*	pBAD18 with fragment containing signal peptide deleted yccT	This study
pBADyccT-FLAG	pBAD18 with fragment containing yccT::3×FLAG	This study
pSU311	Vector containing a 1.9-kb fragment carrying the R6K oriV and the βla gene	[70]
pSUB11	pSU311 with fragment containing 3xFLAG sequence	[70]
pKD46	araC, bla, tL3	[73]
pET-21(a)	Ampr, T7 expression plasmid	Novagen
pET21yccT-His	C-terminal His6 tagged with fragment containing yccT::His-tag	This study
pET21csgA-His	C-terminal His6 tagged with fragment containing csgA::His-tag	This study
pTAKN-2-bsfgfp	pTAKN-2 with fragment containing sfgfp ORF	Gifted by Dr. S. Sugimoto
pBADsfgfp	pBAD24 with fragment containing sfgfp ORF	This study
pBADyccT-sfgfp	pBAD24 with fragment containing yccT ORF and sfgfp ORF	This study
pBADyccT*-sfgfp	pBAD24 with fragment containing dspyccT ORF and sfgfp ORF	This study
Primer	Sequence(5′–3′)	
yccT(fw)3FLAG	AAATACTTTCCTGCAGTGGGCGGAAAAACAACCATCTTCCGACTACAAAGACCATGACGG	
yccT(rv)3FLAG	CCGTAGTTAACTTTCCTACAGATTACTGTAAGCACTTATCCATATGAATATCCTCCTTAG	
yccT-s-N	ATAATGGACCGCATCAGTTAGTGTT	
yccT-t-N	TCAGGAAGATGGTTGTTTTTCCG	
csgD-N-F	TTATCGCCTGAGGTTATCGTTTGC	
csgD-N-R	TCTTCAGGCTCTATTATTCTTCTGGATAT	
csgB-s-N	TTTATGATGTTAACAATACTGGGTGCGC	
csgB-t-N	TTAACGTTGTGTCACGCGAATAGCCATTT	
yccT-PET-NdeI-F	GGAGCTACATATGAAAACCGGCATCGTGAGCACCT	
yccT-PET-NotI-R	GGCACAAGCGGCCGCGGAAGATGGTTGTTTTTCCG	
csgA-NdeI-PETF	GGGGTTTCATATGGGTGTTGTTCCTCAGTACGGCG	
csgA-NotI-PETR	ACAAATGGCGGCCGCGTACTGATGAGCGGTCGCGT	
T7promoterprimer	TAATACGACTCACTATAGGG	
T7terminatorprimer	GCTAGTTATTGCTCAGCGG	
yccT-EcoRI-F	AGTATGAATTCAGAAAAATTTTGACACATT	
yccT-XbaI-R	CAAGATCTAGACCGAAAAAGCCTGCGCACAG	
BAD-SQ-F	CTGTTTCTCCATACCCGTT	
BAD-SQ-R2	TTTCACTTCTGAGTTGGCATGGGGTCAGG	
yccT-FLAG-BAD-F	GACTACAAAGACCAT GTGCCTGTGCGCAGG	
yccT-FLAG-BAD-R	TTTATCGTCGTCATC TGTTTTTCCGCCCAC	
Flag-check-R2	AGCGTGGTCATAGGTAGCTCCTGTCAAAAGACCGC	
yccT-SPdel-F	ACCTATGACCACGCT TTTCAACCGATGTCG	
yccT-SPdel-R	AGCGTGGTCATAGGT CTGTCAAAAGACCGC	
BAD-24-SQ-F	TTTATCCATAAGATTAGCGGATCCTACCTG	
BAD-24-SQ-R	ATCTGTATCAGGCTGAAAATCTTCTCTCAT	
pBAD24-inversePCR-F	TACCCGGGGATCCTCTAGAGTCGACCTGCAGGCAT	
pBAD24-inversePCR-R2	GGTGAATTCCTCCTGCTAGCCCAAAAAAACGGGTA	
yccT-infusion-pBAD24-F2	CAGGAGGAATTCACCATGAAAACCGGCATCGTGAC	
dspyccT-infusion-pBAD24-F2	CAGGAGGAATTCACCATGACCACGCTGCTGCGCGG	
yccT-infusion-R	TTCGCCTTTCGACATGGAAGATGGTTGTTTTTCCG	
sfGFP-infusion-F	AAACAACCATCTTCCATGTCGAAAGGCGAAGAACT	
sfGFP-infusion-p24-F	CAGGAGGAATTCACCATGTCGAAAGGCGAAGAACT	
sfGFP-infusion-R	GAGGATCCCCGGGTATTATACAGCTCATCCATACC	

## Data Availability

All data are presented within the manuscript and the Supplementary Materials.

## Supplementary Materials

The following supporting information can be downloaded at: https://www.mdpi.com/article/10.3390/ijms24054357/s1.
