# Supplementary material for: CsgI (YccT) Is a Novel Inhibitor of Curli Fimbriae Formation in Escherichia coli Preventing CsgA Polymerization and Curli Gene Expression
Source: Int J Mol Sci. 2023 Feb 22;24(5):4357. doi: 10.3390/ijms24054357 (PMC10002515; doi:10.3390/ijms24054357)
Supplement: Supplementary file 1 [file ijms-24-04357-s001.zip › ijms-2165637-supplementary.pdf]

Z-stack images

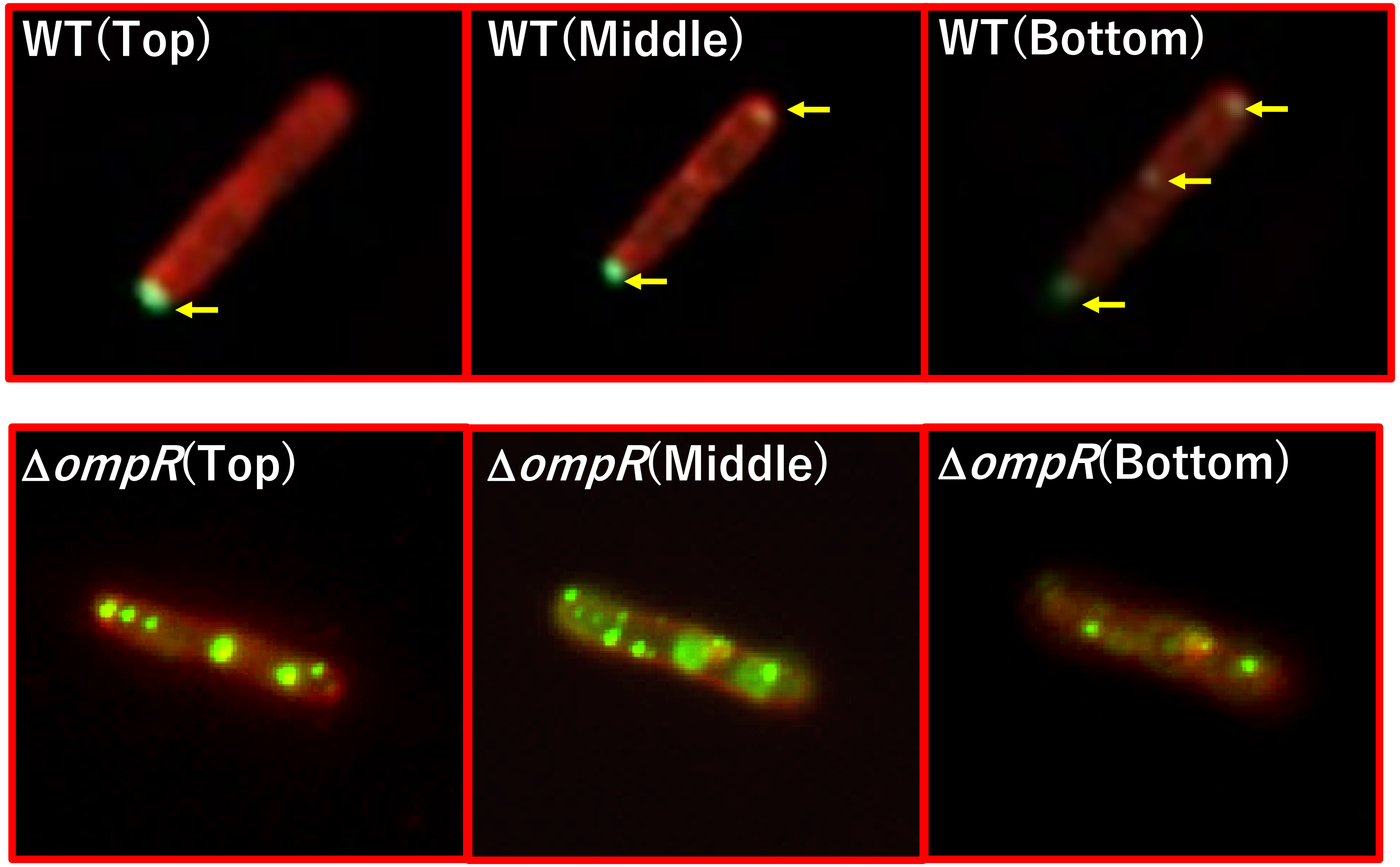

Supplemental Figure S1. Effects of *ompR* deletion on curli fimbriae formation and YccT localization. A wild-type (WT) strain and a strain with an ompR deletion (Δ*ompR)* were transformed with pBADyccT-sfGFP and grown in LB medium in the presence of 0.02% (final concentration) arabinose at 37° C for 2 hours. The membranes of the *E.coli* cells were stained with FM4-64. Various images from the Z-stack (top, middle, and bottom images) of the cells are presented.
